# Ignavine: a novel allosteric modulator of the μ opioid receptor

**DOI:** 10.1038/srep31748

**Published:** 2016-08-17

**Authors:** Katsuya Ohbuchi, Chika Miyagi, Yasuyuki Suzuki, Yasuharu Mizuhara, Keita Mizuno, Yuji Omiya, Masahiro Yamamoto, Eiji Warabi, Yuka Sudo, Akinobu Yokoyama, Kanako Miyano, Takatsugu Hirokawa, Yasuhito Uezono

**Affiliations:** 1 Tsumura Research Laboratories, Tsumura and Co., 3586 Yoshiwara, Ami-machi Inashiki-gun, Ibaraki 300-1192, Japan; 2 Division of Cancer Pathophysiology, National Cancer Center Research Institute, 5-1-1 Tsukiji, Chuo-ku, Tokyo 104-0045, Japan; 3 Environmental Molecular Biology Laboratory, Faculty of Medicine, University of Tsukuba, 1-1-1 Tennodai, Tsukuba-shi, Ibaraki 305-8575, Japan; 4 Molecular Pathology and Metabolic Disease, Faculty of Pharmaceutical Sciences, Tokyo University of Science, 2641 Yamazaki, Noda-shi, Chiba 278-0022, Japan; 5 Molecular Profiling Research Center for Drug Discovery, AIST Tokyo Waterfront Bio-IT Research Building 2-4-7 Aomi, Koto-ku, Tokyo 135-0064, Japan; 6 Dicision of Biomedical Science, Faculty of Medicine, University of Tsukuba, 1-1-1 Tennodai, Tsukuba-shi, Ibaraki 305-8575, Japan; 7 Division of Supportive Care Research, National Cancer Center Research Institute, 5-1-1 Tsukiji, Chuo-ku, Tokyo 104-0045, Japan

## Abstract

Processed *Aconiti* tuber (PAT) is used to treat pain associated with various disorders. Although it has been demonstrated that the κ opioid receptor (KOR) signaling pathway is a mediator of the analgesic effect of PAT, active components affecting opioid signaling have not yet been identified. In this study, we explored candidate components of PAT by pharmacokinetic analysis and identified ignavine, which is a different structure from aconitine alkaloids. A receptor binding assay of opioid receptors showed that ignavine specifically binds the μ opioid receptor (MOR), not the KOR. Receptor internalization assay in MOR-expressing cell lines revealed that ignavine augmented the responses produced by D-Ala(2)-N-Me-Phe(4)-Gly-ol(5)-enkephalin (DAMGO), a representative MOR agonist, at a low concentration and inhibited it at a higher concentration. Ignavine also exerted positive modulatory activity for DAMGO, endomorphin-1 and morphine in cAMP assay. Additionally, ignavine alone showed an analgesic effect *in vivo. In silico* simulation analysis suggested that ignavine would induce a unique structural change distinguished from those induced by a representative MOR agonist and antagonist. These data collectively suggest the possibility that ignavine could be a novel allosteric modulator of the MOR. The present results may open the way for the development of a novel pain management strategy.

The tuber of the species *Aconitum* is a crude drug that has been utilized from ancient times. Since the *Aconiti* tuber has toxic ingredients, attempts to reduce its toxicity have been made by heating. The processed *Aconiti* tuber (PAT) is used in Japan to treat pain associated with various disorders as an important component of Japanese traditional medicines (Kampo) such as Goshajinkigan (GJG) and Keishikajutsubuto. It has been reported that PAT exhibits pharmacological effects such as analgesia^1^,^2^,^3^,^4^, peripheral vasodilation^5^,^6^, anti-inflammatory^7^ and anti-pollakiuria^8^ effects in rodent models. In the clinic, the analgesic effect of Keishikajutsubuto was enhanced by the addition of PAT^9^.

Regarding the analgesic effect, PAT showed beneficial effects in repeatedly cold-stressed mice^3^ and in a rat model of chronic constriction injury (CCI)^2^. It has been reported that the analgesic effects of PAT can be blocked by anti-dynorphin antibody^10^ and by nor-binaltorphimine (nor-BNI)^2^, a specific antagonist of the κ opioid receptor (KOR). In addition, PAT bas been shown to ameliorate morphine tolerance and this effect was also blocked by nor-BNI^11^,^12^. These results suggested that these pharmacological effects must be mediated by direct or indirect activation of the KOR.

On the other hand, no active ingredients which affect the dynorphin-KOR system have been identified. Although it is well known that aconitine alkaloids in PAT such as mesaconitine^13^, aconitine^14^ and benzoylmesaconine^15^ have analgesic activity, it remains unclear whether these aconitine alkaloids act on the opioid systems. It has been noted that the content of diester-type alkaloids such as mesaconitine and aconitine are converted to monoester alkaloids such as benzoylmesaconine and benzoylhypaconine by heating the *Aconiti* tuber. The analgesic effect of PAT was shown to be comparable to that of the unprocessed *Aconit*i tuber in a mouse model of repeated cold stress^1^. Additionally, the analgesic activity of benzoylmesaconine, the main component of PAT, would not be enough to fully account for the analgesic effect of PAT^15^. Therefore it is likely that there are other unknown ingredients contributing to the analgesic effect.

In the present study, candidate ingredients were identified by performing a pharmacokinetic study of GJG, which was found to be composed of ten crude drugs including PAT and to have analgesic activity^16^,^17^ (Supplementary Figure S1). Ignavine was also detected in the plasma of GJG-treated rats. Characterization *in vitro*, *in vivo* and *in silico* revealed that ignavine has a unique character as a μ opioid receptor (MOR) modulator with analgesic activity.

## Results

### Pharmacokinetic study of the PAT-containing traditional medicine, GJG

**Figure 1:**
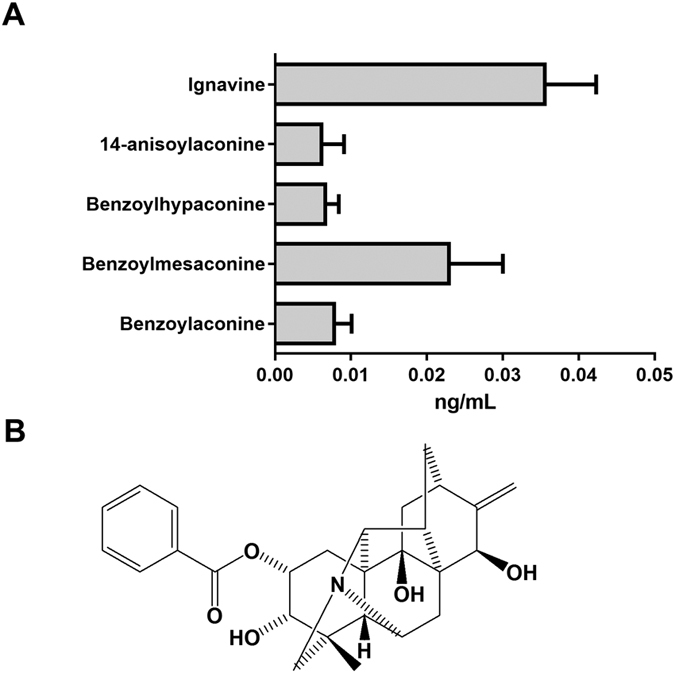
Rat pharmacokinetic study of GJG and structure of ignavine. (**A**) A dose of 1 g/kg GJG was orally administered and blood samples were collected 1 h after administration. Quantitative analysis of the ingredients in PAT was performed using LC/MS/MS. Data are expressed as mean +/− SD (n = 5). (**B**) Ignavine is a diterpene alkaloid and has a different core structure from the aconitine alkaloids.

A pharmacokinetic study of GJG was performed to identify the bioavailable molecules in PAT. GJG (1 g/kg) was orally administered to rats and blood samples were collected 1 h after administration. Then, representative components of PAT in the collected blood samples were measured by LC/MS/MS. As shown in Fig. 1A, higher concentrations of ignavine (Fig. 1B) and of benzoylmesaconine were detected among the measured ingredients. Therefore, we further investigated whether ignavine and benzoylmesaconine affect the opioid system.

### Receptor binding assay for opioid receptors

**Figure 2:**
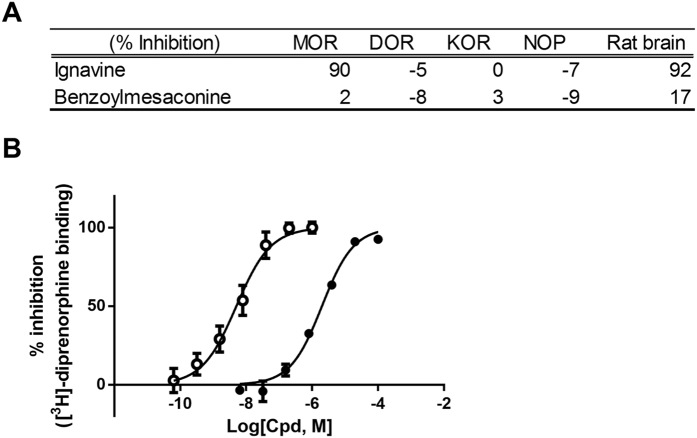
Binding affinity of the components of opioid receptors. (**A**) Inhibition of competitive radioligand binding to the human MOR- ([^3^H]-diprenorphine), DOR- ([^3^H]-naltorindole), KOR- ([^3^H]-diprenorphine), and NOP- ([^3^H]-nocicepine) expressing cell lines and rat whole brain ([^3^H]-naloxone). Concentration of test compounds was 10 μM. Data are represented as mean (n = 2). (**B**) Concentration-dependent inhibition of [^3^H]-diprenorphine binding against the MOR. Open circles and closed circles represent naloxone and ignavine respectively. IC_50_ values of naloxone and ignavine were 5.1 nM and 2.0 μM respectively. Data are represented as mean +/− SEM (n = 3).

First, the receptor binding assay was performed using membrane fractions prepared from human MOR-, δ opioid receptor (DOR)-, KOR-, and nociception receptor (NOP)-expressing cell lines and from rat brain for evaluation of non-specific binding. The % inhibition at a concentration of 10 μM is shown in Fig. 2A. Neither ignavine nor benzoylmesaconine inhibited the binding of radio-labeled ligand ([^3^H]-diprenorphine) to KOR, which was thought to be involved in the analgesic effect of PAT. On the other hand, unexpectedly, ignavine almost completely inhibited [^3^H]-diprenorphine binding to an MOR-expressing membrane. Ignavine also inhibited its binding in the membrane fraction from rat brain. Dose titration analysis of ignavine showed that its IC_50_ value was 2.0 μM (Fig. 2B).

### Receptor internalization assay of ignavine

**Figure 3:**
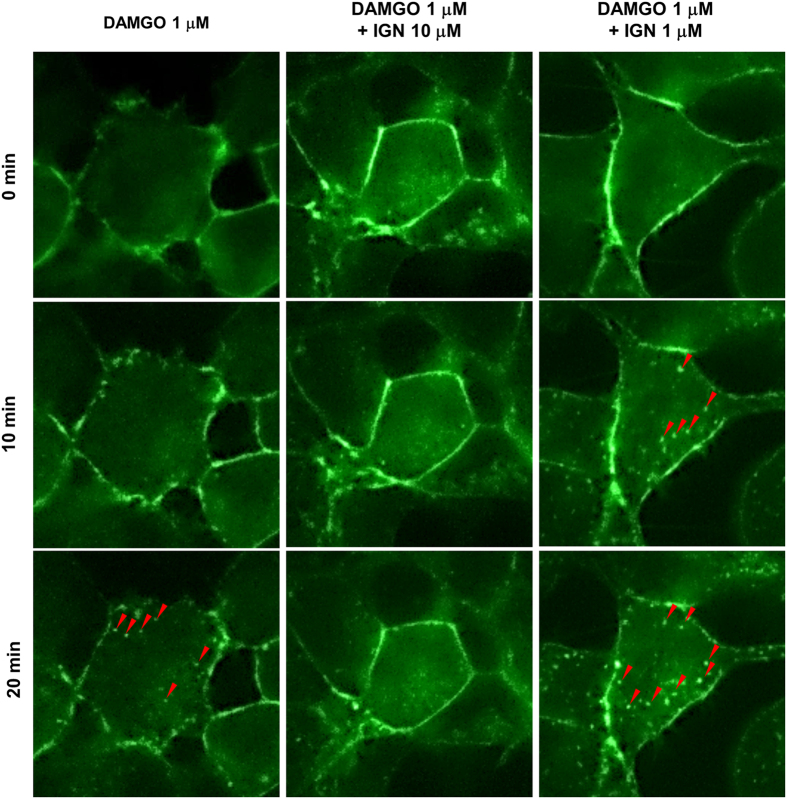
Receptor internalization of MOR. MOR-GFP-expressing HEK-293 cells were treated with DAMGO and/or ignavine (IGN). DAMGO induced vesicle formation (shown by red arrowheads) in the intracellular space, which represented receptor internalization, 20 min after administration. A concentration of 10 μM ignavine blocked internalization. In contrast, 1 μM ignavine accelerated DAMGO-induced receptor internalization.

To clarify whether ignavine was an agonist or antagonist of MOR, a receptor internalization assay was performed using GFP-tagged MOR-expressing HEK-293 cells. Photomicrographs of representative cells are shown in Fig. 3 and all field views of cells treated with 1 μM D-Ala(2)-N-Me-Phe(4)-Gly-ol(5)-enkephalin (DAMGO) and 1 μM DAMGO with 1 μM ignavine are shown in Supplementary Figure S2. As shown in Fig. 3, fluorescence was mainly detected on the surface of the cell membrane (0 min in Fig. 3). Twenty minutes after treatment with 1 μM DAMGO, vesicles were observed in the intracellular space, indicating receptor internalization (Fig. 3, shown by red arrow-head). Treatment with 10 μM ignavine alone did not affect receptor internalization (data not shown), but co-treatment blocked internalization induced by DAMGO (Fig. 3). In contrast, co-treatment with 1 μM ignavine and 1 μM DAMGO induced MOR internalization 10 min after treatment and vesicle formation was enhanced compared to that with DAMGO alone. Taken together, these results suggest that ignavine can modulate MOR activity and that the mode of modulation differs depending on its concentration.

### Intracellular cAMP assay of ignavine

**Figure 4:**
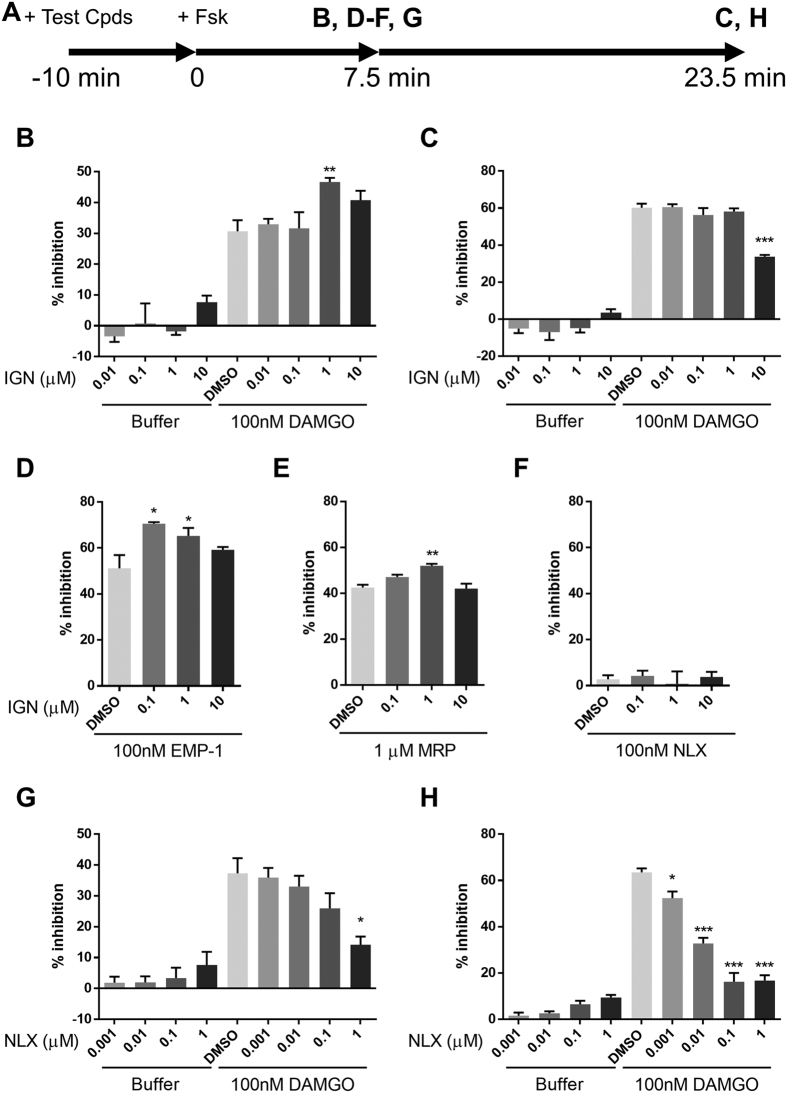
Intracellular cAMP assay. Intracellular cAMP levels were evaluated using the recombinant cell line stably expressing the MOR and cAMP biosensor protein. (**A**) Test compounds (ignavine (IGN), naloxone (NLX) + DAMGO, endomorphin-1 (EMP-1), morphine (MRP) or NLX) were added 10 min before forskolin (Fsk) treatment. Then, luminescence intensity was measured at 7.5 min (**B**,**D**–**F**,**G**) and 23.5 min (**C**,**H**). Ignavine significantly inhibited MOR activation by DAMGO in the late phase (**C**), but in contrast, enhanced the activation of DAMGO in the early phase (**B**). Ignavine also showed positive modulation of EMP-1 (**D**) and MRP (**E**), but not NLX (**F**), at 7.5 min after Fsk stimulation. NLX inhibited MOR activation by DAMGO during both phases (**D**,**E**). Data are expressed as mean +/− SEM (n = 3–6). *P < 0.05, **P < 0.01, ***P < 0.001 by Dunnett’s test vs. DMSO - 100 nM DAMGO.

G protein-coupled receptors activate two directional signaling pathways. One is via β-arrestin which leads to induction of receptor internalization; the other is through second messengers such as cAMP and Ca^2+^. Since MOR belongs to the Gi-coupled family of receptors, activation of MOR leads to a decrease in intracellular cAMP level. We next investigated the effect of ignavine on intracellular cAMP content. Intracellular cAMP was measured using a biosensor protein, GloSensor™ (Promega, Madison, WI, USA). This technology enables kinetic measurement of cAMP^18^. The experimental design is shown in Fig. 4A. Cells were simultaneously treated with ignavine and DAMGO 10 min before stimulation with forskolin, then the amount of intracellular cAMP was traced by luminescence intensity. The results showed that DAMGO inhibited the increase of intracellular cAMP induced by forskolin (Supplementary Figure S3). Ignavine alone showed no effect on intracellular cAMP levels either 7.5 min or 23.5 min after forskolin treatment (Fig. 4B,C). However, when added together with 100 nM DAMGO, ignavine showed an intriguing characteristic, with a concentration of 1 μM enhancing the activity of DAMGO in the early period (Fig. 4B) but a concentration of 10 μM inhibiting DAMGO activity in the later period (Fig. 4C). These data are in good accordance with the results of the receptor internalization assay. In addition to DAMGO, other MOR agonists and antagonists, endomorphin-1, morphine and naloxone were evaluated in this assay. Ignavine also enhanced the activity of these endogenous and exogenous agonists 7.5 min after forskolin stimulation (Fig. 4D,E) and inhibited them 23.5 min after stimulation (Supplementary Figure S4). On the other hand, ignavine did not show a clear response in the presence of naloxone (Fig. 4F and Supplementary Figure S4).

We next investigated whether or not a representative MOR antagonist, naloxone, had similar characteristics to ignavine. As shown in Fig. 4G,H, naloxone blocked the activity of DAMGO at both time points. These results indicate that ignavine has a unique character different from that of the typical MOR antagonist.

**Figure 5:**
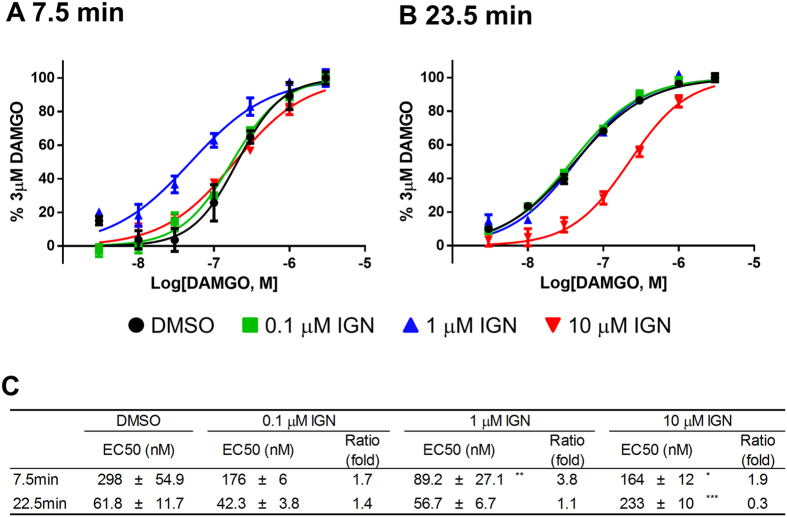
Dose-response curve in intracellular cAMP assays. (**A**,**B**) Activation curves show representative data from three independent experiments. In the early phase (7.5 min after forskolin administration), ignavine shifted the activation curve of DAMGO to the left, which indicated that ignavine worked as an MOR enhancer (**A**). In contrast, in the late phase (23.5 min after forskolin administration), ignavine shifted the activation curve to the right, which indicated that ignavine acted as an MOR inhibitor (**B**). EC_50_ values of DAMGO and Ratio (fold-change of EC_50_) are shown in (**C**). Data shown represent the mean +/− SEM (n = 3). *P < 0.05; **P < 0.01; ***P < 0.001 by Dunnett’s test vs. DMSO.

Figure 5 shows the effect of ignavine on the dose titration curve and EC_50_ values of DAMGO. In agreement with the previous results, ignavine produced a leftward shift in response to DAMGO at 7.5 min after forskolin treatment (Fig. 5A) and a rightward shift at 23.5 min (Fig. 5B). EC_50_ values were significantly shifted, with a 3.8-fold decrease caused by 1 μM ignavine at 7.5 min and a 3.3-fold increase with 10 μM ignavine at 23.5 min (Fig. 5C).

### *In vivo* analgesia study of ignavine

**Figure 6:**
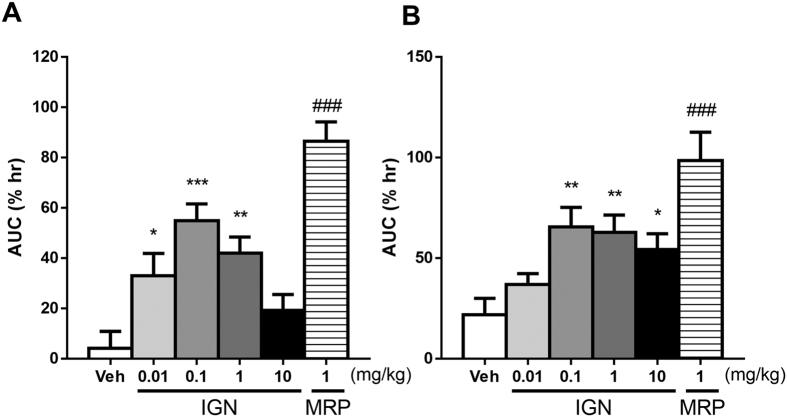
Effects of ignavine on antinociception assessed by the tail-flick test (**A**) and tail-pressure test (**B**). Ignavine (IGN) and morphine (MRP) were intra-peritoneally or subcutaneously administered, respectively. Responses to each stimulant were measured at 30, 60, 90, 120 and 180 min after administration. Analgesic activity is represented by the area under the curve. Data represent the mean +/− SEM (n = 10–11). *P < 0.05; **P < 0.01; ***P < 0.001 by Dunnett’s test vs. vehicle. ^###^*P* < 0.001 by Student’s *t*-test vs. vehicle.

Analgesic activity of ignavine was investigated by the tail-flick (Fig. 6A) and tail-pressure tests in normal mice (Fig. 6B). Intra-peritoneal administration of ignavine inhibited pain in both tests. Maximum activity was shown at a dose of 0.1 mg/kg and analgesic activity was attenuated at higher doses. These dose-dependent relationships were similar to the results of *in vitro* assays.

### *In silico* simulation of the interaction between ignavine and the MOR

**Figure 7:**
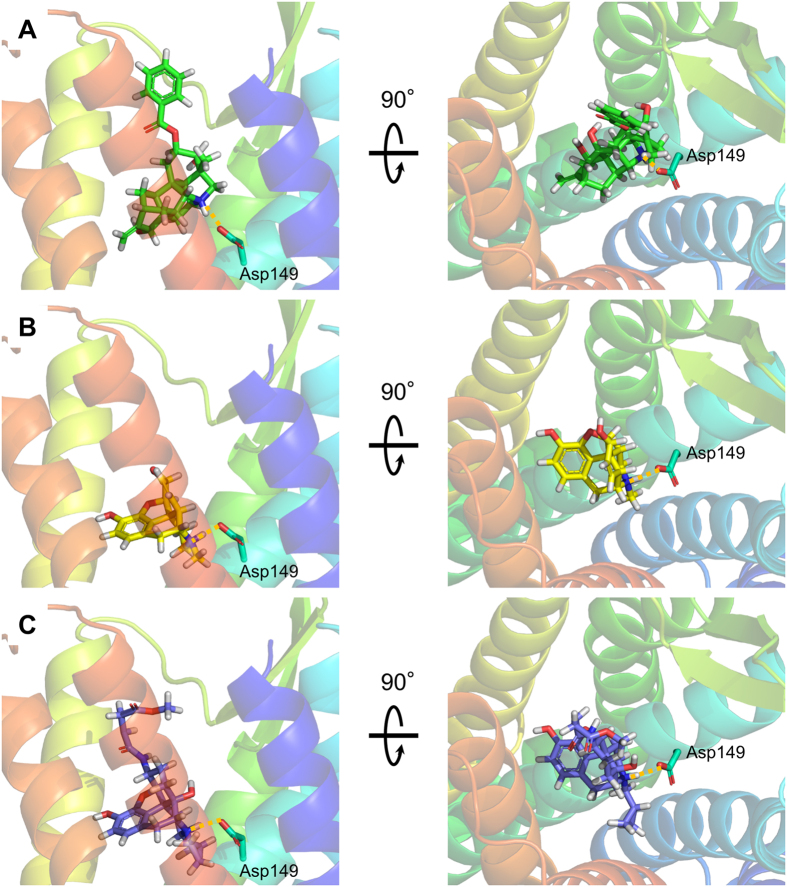
Predicted interactions between ligands and a human MOR homology model viewed from the extracellular side (left panel) and parallel to the membrane (right panel). Ignavine (**A**), morphine (**B**) and β-FNA (**C**) all have a quaternary amine structure, which interacts with Asp149. This interaction is shown by a yellow dotted line.

**Figure 8:**
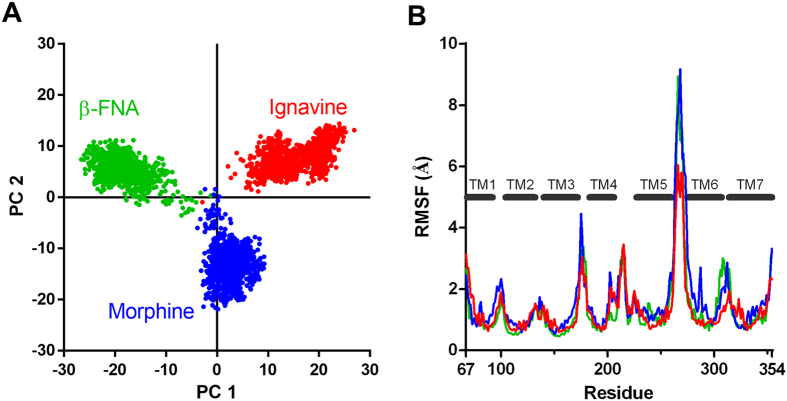
Molecular dynamics simulation of the ignavine–human MOR complex. (**A**) Two-dimensional principal component analysis (PCA) projections of trajectories obtained from MD simulations. (**B**) Root mean square fluctuations (RMSF) of each amino acid residue in the human MOR molecule during MD simulation. Data of ignavine, morphine and β-FNA are represented in red, blue and green, respectively.

Finally, we performed a computational simulation study to investigate in detail the interaction between ignavine and MOR protein. A human MOR homology model was constructed using the crystal structure of mouse MOR^19^ as a template (Supplementary Figure S5). Morphine and β-funaltrexamine (β-FNA) were used for the simulation study as a representative MOR agonist and antagonist, respectively, and we constructed docking models of each ligand (Fig. 7). The result of docking simulation suggests that ignavine could also bind MOR at the orthosteric binding site. As shown in the constructed docking model, all ligands tested here showed a hydrophilic interaction with Asp149 in the human MOR. By using these docking models, we conducted computational analysis of MD simulation over 100 nsec. To estimate the effect of each ligand on the MOR structure during MD simulation, we performed Principal Component Analysis (PCA), one of the methods of multivariate analysis used for analyzing the net effect of phenomena consisting of numerous factors, of the structural coordinate of each MOR–ligand complex. By using the root mean square deviation (RMSD) distance data for Cα atoms of the transmembrane region in human MOR models obtained from this computation, each trajectory data-set was plotted in accordance with principal components 1 and 2 for abscissa and ordinate, respectively. As shown in Fig. 8A, the three ligands tested here could be clearly distinguished in a 2-dimensional scatterplot of PCA. In addition to PCA analysis, a spatial fluctuation map, which represents the conformational flexibility during MD simulation, is shown in Fig. 8B. The flexibility of the loop structure between TM5 and TM6 in our ignavine-docking model was smaller than those in morphine- and β-FNA-docking models. These simulation data suggest that the structural change caused by ignavine binding would be different to that caused by either the reference agonist (morphine) or the antagonist (β-FNA).

## Discussion

In this study, we explored the candidate components which were expected to play a role in analgesic activity of PAT. From our data we identified ignavine and found this candidate molecule to affect MOR activity in a unique manner and to exert analgesic activity in mice.

A pharmacokinetic study of GJG showed that ignavine and benzoylmesaconine could be detected in the plasma 1 hour after administration (Fig. 1A). Benzoylmesaconine is known to transit to the systemic blood circulation after oral administration of either benzoylmesaconine alone or of PAT-containing prescriptions^20^,^21^. The result shown in this study is in line with previous reports. Although benzoylmesaconine has analgesic activity itself^15^, it does not interact with opioid receptors according to the results of our binding assay (Fig. 2). Therefore, benzoylmesaconine must act to suppress pain independent of opioid signaling. On the other hand, ignavine was detected at the highest concentration among the measured components. This is the first report to show that ignavine could be absorbed into the systemic blood. Ignavine is a diterpene alkaloid, with a different structure from the aconitine alkaloids, and is a component of *Aconitum* tuber^22^ and PAT^23^. Ignavine has been reported to be one of the low-toxicity components of *Aconitum* tuber and to have anti-inflammatory activity in the acetic acid-induced writhing and carrageenan paw edema test without any adverse effects^24^. However, no target molecules of ignavine have been identified to date. In this study, using the receptor binding assay, we found that ignavine specifically interacts with MOR.

An *in vitro* functional assay revealed that ignavine exhibited bidirectional activity in a concentration-dependent manner. At high concentrations (>10 μM), ignavine inhibited MOR activity both in a receptor internalization assay and in an intracellular cAMP assay (Figs 3 and 4). It has been reported that high doses of PAT inhibit the antinociception induced by morphine in mice^25^. The inhibitory activity of ignavine might therefore contribute to this phenotype. In contrast, enhancement of MOR activity was observed at low concentrations of ignavine (~1 μM). In the tail-flick test, 0.1 mg/kg ignavine showed the maximum response, while higher concentrations of ignavine weakened analgesic activity (Fig. 6A). The tail pressure test also showed the same tendency towards dose dependence. These bell-shaped dose-dependency curves observed *in vivo* were in good accord with the *in vitro* profiles of ignavine. Since ignavine was shown to enhance the activity of the endogenous MOR ligand, endomorphin-1 (Fig. 4D), it is possible that it may exert analgesic activity by single administration *in vivo*. Therefore, it can be presumed that the interaction of ignavine with the MOR might be involved in analgesia in these mice.

Ignavine positively modulated the response of DAMGO at a low concentration (~1 μM). In the present study, ignavine accelerated the receptor internalization triggered by DAMGO (Fig. 3) and enhanced the potency of several MOR agonists in the intracellular cAMP assay (Fig. 4) without affecting MOR activity when used alone. These features are comparable to those of the positive allosteric modulator, BMS-986122, reported by Burford *et al.*^26^. Although the binding site of BMS-986122 is different from that of ignavine (ignavine binds the MOR orthosterically (see below)), BMS-986122 showed positive modulation of endomorphin-I both in β-arrestin recruitment and in intracellular cAMP assay. Since positive modulators of MOR are expected to maintain the temporal and spatial fidelity of native signaling, they are proposed to avoid on-target side effects of exogenous MOR agonists such as respiratory suppression, constipation, allodynia, tolerance, dependence and withdrawal symptoms^27^. Our results showed that a single administration of ignavine exerted an analgesic effect in mice. Therefore, ignavine is also likely to be a promising agent for analgesia. A long-term dosing study should be performed to investigate any adverse effects of ignavine.

Regarding the binding site of ignavine, the receptor binding assay showed that ignavine inhibited the binding of [^3^H]-diprenorphine, which has the same structure as morphine and β-FNA. The docking simulation supports the result of the binding assay. Like morphine and β-FNA, ignavine has a quaternary amine structure. This structure has been reported to interact with Asp147 of the rat MOR (Asp149 in the human MOR as shown in Fig. 7) and this interaction is important for the pharmacological properties of morphine^19^,^28^. Taken together with these results, these data strongly suggest that ignavine directly interacts with the MOR at the orthosteric binding site.

Additionally, MD simulation of an ignavine-MOR complex model indicates that orthosteric binding of ignavine induces structural change in a unique manner (Fig. 8). PCA analysis of MD simulation clearly distinguishes the trajectory of conformation change induced by ignavine from those brought about by a representative MOR agonist and antagonist. Since the PC-1 axis divides the three compounds, the difference might reflect the unique character of ignavine. On the PC-2 axis, the plot area of ignavine overlapped that of β-FNA and was separate from that of morphine, indicating that the PC-2 axis might differentiate between agonist and antagonist.

Docking simulation indicates that a single MOR binding pocket does not have enough capacity to accommodate both ignavine and an agonist simultaneously. Since opioid receptors including MOR can form homo- and hetero-dimers in recombinant-expressing cells^29^, these dimeric opioid receptors would have two binding pockets. The binding assay clearly showed that ignavine is an orthosteric ligand for MOR. Therefore, the excess amount of ignavine should occupy both binding pockets in an MOR dimer as suggested in Fig. 2B (high concentrations of ignavine completely inhibited the binding of [^3^H]-diprenorphine). However, if the concentration of ignavine was not sufficient, ignavine would not occupy both pockets, leaving one of the pockets vacant. Because the low and high concentrations of ignavine increased and decreased the activity of agonists, respectively, we propose the following hypothesis: when ignavine and another agonistic ligand (such as DAMGO, morphine, or endogenous endomorphin-1) occupy each binding pocket in a homodimer, ignavine might exert positive modulatory activity. On the other hand, ignavine at higher concentration would bind to both binding pockets in a homodimer, exerting antagonistic activity. It has been reported that DOR ligands can enhance DAMGO binding capacity in a μ-δ heteromer^30^. It is hypothesized that DOR ligands bind the DOR of the heteromer and “allosterically” modulate the activity or binding capacity of the partner receptor. Ignavine is also expected to exert an enhancing effect on activity by a similar mechanism. Smith *et al.* named the allosteric modulation mentioned above as “off-target allosterism”, meaning allosteric modulation of another binding site (orthosteric or allosteric) on a distinct protein such as a dimeric partner^31^. Therefore ignavine could be defined as an “off-target allosteric modulator”.

Since PAT can produce analgesia directly or indirectly through the KOR and/or induction of dynorphin release^2^,^10^, it is possible that ignavine might contribute to the analgesic activity of PAT by modulating μ-κ heteromer signaling. To test this hypothesis, it will be necessary to evaluate the effect of ignavine on μ-κ heteromer signaling. We plan to carry out the characterization of ignavine’s activity with heteromers including μ-κ heteromer.

In conclusion, we identified ignavine and demonstrated the possibility that ignavine produces analgesia via positive modulation of MOR signaling. Pain management using a positive modulator of opioid receptors is an attractive approach to avoid the serious adverse effects of conventional opioid drugs. It is expected that ignavine could be a candidate compound to evaluate this promising strategy.

## Materials

Goshajinkigan, benzoylmesaconine and ignavine were supplied by Tsumura (Tokyo, Japan). DAMGO, naloxone, forskoline, 3-isobutyl-1-methylxanthine (IBMX) and Ro 20-1724 were purchased from Sigma Aldrich (St Louis, MO, USA). Morphine hydrochloride was purchased from Takeda Pharmaceutical Co., Ltd. (Tokyo, Japan).

### Rat pharmacokinetic study

All experimental procedures were ethically approved by the Laboratory Animal Committee of Tsumura and Co. and performed according to the institutional guidelines for the care and use of laboratory animals, which is in accordance with the National Institutes of Health Guide for the Care and Use of Laboratory Animals.

Male Sprague-Dawley rats (7 weeks, Japan Clea Inc., Tokyo, Japan) were fasted for more than 16 hours with access to water. At 1 h after oral administration of GJG (1.0 g/10 mL/kg, n = 5), blood samples were collected with a heparin-coated syringe and centrifuged at 1,500 × *g* for 15 minutes at 4 °C. The plasma samples were stored at −80 °C until use.

Plasma samples were pretreated by solid-phase extraction (Oasis^®^ MCX μ Elution Plate, 30 μm; Waters Corporation, Milford, MA, USA) and injected into the LC/MS/MS system for analysis. Components of GJG in plasma were measured by a QTRAP^®^5500 system (AB SCIEX, Framingham, MA, USA) equipped with an Agilent 1260 HPLC system (binary pump, online degasser, auto plate-sampler and column oven; Agilent Technologies, Santa Clara, CA, USA). The lower limits for quantification were 0.01 ng/mL for ignavine; 0.002 ng/mL for benzoylaconine; 0.001 ng/mL for benzoylmesaconine, benzoylhypaconine and 14-anisoylaconine.

### Plasmid constructs and transfection

Human MOR cDNAs (NM_000914) with or without a stop codon were amplified from the Flexi ORF clone (Promega, Madison, WI, USA). The amplified human MOR fragment was introduced into the pcDNA3.1 (+) vector (Life Technologies, Carlsbad, CA, USA). Additionally, the GFP coding sequence was inserted into the 3′ terminus of the stop codon-deleted human MOR sequence. Using these constructed plasmids, HEK-293 cells stably expressing the human MOR or human MOR-GFP complex were generated using Lipofectamine reagent (Life Technologies) and selected by MOR activity. Regarding non-tagged human MOR-expressing HEK-293 cells, pGloSensor™-22F plasmid (Promega) was additionally introduced into the cells and stable cell lines were generated.

### Binding assay

The receptor binding assay for primary screening was performed by MDS Pharma Services (King of Prussia, PA, USA). Assays were performed under the conditions indicated in Table 1. A dose titration study was performed using human MOR-expressing HEK-293 cells established in this study. Whole cell fractions were incubated with [^3^H]-diprenorphine (Perkin Elmer, Waltham, MA, USA) in the presence of varying concentrations of ignavine or naloxone. After 1 h, assays were harvested onto GF/C filtermats using a Filtermate harvester (Perkin Elmer). Then, MeltiLex scintillant (Perkin Elmer) was melted onto dried filtermats and the residual bound radioligand was measured by scintillation counting in a TriLux microbeta counter (Perkin Elmer). The result of the binding assay was expressed as % inhibition, where % inhibition = 100 × (1−((CPMtest−CPMnsb)/(CPMtb−CPMnsb))), CPM: Count per minute, CPMtest: CPM obtained in the presence of test compound, CPMnsb: CPM obtained in the presence of non-specific ligand, CPMtb: CPM obtained without cold compound.

### Receptor internalization assay

Internalization assay was performed using the HEK-293 cells stably expressing the human MOR-GFP complex. When the GPCR is activated and internalized, the receptor is observed inside vesicle-like structures within the cytoplasm. This phenomenon was evaluated by imaging the fluorescence of the GFP-tag, which revealed translocation of the receptor from the plasma membrane to intracellular vesicles. Cells were seeded into a four-compartment cell culture dish at 1.5 × 10^5 ^cells/compartment in DMEM supplemented with 10% fetal bovine serum. The next day, the culture medium was changed to assay buffer (20 mM HEPES, 115 mM NaCl, 5.4 mM KCl, 0.8 mM MgCl_2_, 1.8 mM CaCl_2_ and 13.8 mM D-glucose at pH 7.4), and incubation was performed at room temperature. Cells were then treated with ignavine, DAMGO or co-treated with ignavine and DAMGO. Fluorescence images of these treated groups were obtained with the same timing using a fluorescence microscope BZ-9000 (Keyence, Tokyo, Japan).

### Intracellular cAMP assay

The GloSensor™ cAMP biosensor (Promega) uses a modified form of firefly luciferase containing a cAMP-binding motif. Upon cAMP binding, a conformational change leads to enzyme complementation, and incubation with luciferase substrate then results in a luminescence readout^18^. Analysis of cAMP accumulation was performed in the HEK-293 cells stably expressing Glosensor™ 22F protein and the human MOR. The cells were seeded into poly-D-lysine-coated 96-well white, clear-bottomed plates (Corning, Corning, NY, USA) at 3 × 10^4^ cells/well in DMEM supplemented with 10% fetal bovine serum. The next day, intracellular cAMP assay was performed using the GloSensor™ reagent following the supplied instructions. Cells were incubated with diluted GloSensor™ reagent at room temperature for 2 h, then stimulated with a test compound (ignavine or naloxone) and DAMGO in assay buffer with 250 μM IBMX and 50 μM Ro 20–1724 for 10 min, followed by the addition of forskolin. Changes in luminescence were detected using FlexStation 3 (Molecular Devices, Sunnyvale, CA, USA). The baseline level of luminescence was measured before test compound application. The response was expressed as % inhibition, which represented the percentage inhibition of forskolin-stimulated cells, or the percentage responses of 3 μM DAMGO-treated cells in each test compound treatment group. Concentration–response curves were fitted using Graph Pad Prism software (Graphpad Software, La Jolla, CA, USA) with a Hill equation model.

### Mouse nociception test

Antinociceptive responses were evaluated by the tail-flick test (Tail flick unit; Ugo Basile, Comerio, Italy) and the tail-pressure test (Pressure analgesimeter; Ugo Basile). The latency (sec) or weight (g) at which mice struggled or withdrew was considered the nociceptive threshold. The antinociceptive activities were calculated as the area under the time exposure curve (AUC) by plotting the percentage increase in the nociceptive threshold (100 × (post-drug nociceptive threshold/pre-drug nociceptive threshold)−100) on the ordinate and the time interval (min) on the abscissa.

### Homology modeling, ligand docking and MD simulation

Homology modeling of the human MOR was conducted using the structure of the mouse MOR complex with β-FNA (PDBID: 4DKL) using Prime (Schrödinger LLC, New York, NY, USA). The third intracellular loop was truncated by the five residues leading out of helix V and the five residues leading into helix VI because it is very long and predicted to be disordered. Initial coordinates of ignavine and morphine were derived from the PubChem database. Energy minimization of ignavine and morphine were performed by the OPLS-AA force field in the LigPrep in Maestro (Schrödinger LLC). These minimized structures were employed as input structures for docking simulations. The human MOR structure was prepared for docking simulations using the Protein Preparation Wizard Script within Maestro. Docking simulations were performed using the Glide SP docking program (Schrödinger LLC). The hydrogen-bonding constraint between the side chain COO- group of Asp 149 was introduced because this hydrogen-bonding formation is highly conserved in almost all known complexes of the Class A GPCR family bound to a wide variety of agonists or antagonists. All of the docked structures were then ranked according to GlideScore. The best position for each docked ligand was manually selected based on both the GlideScore and similarity of the protein–ligand interaction fingerprint to the MOR-β-FNA complex.

Ligand-bound model structures of human MOR were subjected to 100 ns MD simulations using Desmond version 2.3^32^. The OPLS2005 force field was used for the simulations. Initial model structures were placed into a large palmitoyloleoylphosphatidylcholine (POPC) bilayer and TIP3P water molecules solvated with 0.15 M NaCl. After minimization and relaxation of the model, the production MD phase was performed for 50 ns in the isothermal–isobaric (NPT) ensemble at 300 K and 1 bar using Langevin dynamics. Long-range electrostatic interactions were computed using the Smooth Particle Mesh Ewald method. All system setups were performed using Maestro.

### Principal Component Analysis

Principal component analysis (PCA) was performed on the 3D coordinates of Cα atoms of the transmembrane region in the MOR docking models for MD trajectories. The PCA results of three docking structures were analyzed, and the conformational differences were visualized by plotting to PC1 (primary component 1) and PC2 axes. PCA is a standard statistical method routinely used to identify variable correlations in a system from atomic fluctuations in an MD trajectory. The distance matrix of root mean square deviation (RMSD) of the MOR Cα atoms for the combined trajectories of three docking structures was calculated for preparation of input matrix to PCA. PCA was performed using R (version 3.1.1.) which is a software language and environment for statistical computing and graphics.

### Statistical analysis

All values are expressed as means +/− SEM or SD. The statistical significance was evaluated by one- or two-way analysis of variance (ANOVA) followed by Dunnett’s test or Student’s *t*-test. A probability of less than 0.05 was considered significant.

## Additional Information

**How to cite this article**: Ohbuchi, K. *et al.* Ignavine: a novel allosteric modulator of the µ opioid receptor. *Sci. Rep.* **6**, 31748; doi: 10.1038/srep31748 (2016).

## Supplementary Material

Supplementary Information

## Figures and Tables

**Table 1:** Assay condition of the receptor binding assay.

	Ligand	K_d_	B_max_	Non-specific ligand
MOR	Diprenorphine	0.41 nM	3.8 pmol/mg	10 μM Naloxone
KOR	Diprenorphine	0.4 nM	1.1 pmol/mg	10 μM Naloxone
DOR	Naltrindole	0.49 nM	8.6 pmol/mg	10 μM Naloxone
NOP	Nociceptin	1 nM	4.6 pmol/mg	1 μM Orphanin-FQ
Rat brain	Naloxone	1.4 nM	0.095 pmol/mg	1 μM Naloxone
