# Supplementary material for: Ignavine: a novel allosteric modulator of the μ opioid receptor
Source: Sci Rep. 2016 Aug 17;6:31748. doi: 10.1038/srep31748 (PMC4987652; doi:10.1038/srep31748)
Supplement: Supplementary Information [file srep31748-s1.pdf]

Supplementary Figures

Ignavine: a novel allosteric modulator of the μ opioid receptor

Katsuya Ohbuchi[1, 2, *], Chika Miyagi[1, 2], Yasuyuki Suzuki[1], Yasuharu Mizuhara[1], Keita Mizuno[1], Yuji Omiya[1], Masahiro Yamamoto[1], Eiji Warabi[3], Yuka Sudo[2, 4], Akinobu Yokoyama[2, 4], Kanako Miyano[2], Takatsugu Hirokawa[5], Yasuhito Uezono[2, 6]

1) Tsumura Research Laboratories, Tsumura and Co., 3586 Yoshiwara, Ami-machi Inashiki-gun, Ibaraki 300-1192, Japan
2) Division of Cancer Pathophysiology, National Cancer Center Research Institute, 5-1-1 Tsukiji, Chuo-ku, Tokyo 104-0045, Japan.
3) Environmental Molecular Biology Laboratory, Faculty of Medicine, University of Tsukuba, 1-1-1 Tennodai, Tsukuba-shi, Ibaraki 305-8575, Japan.
4) Molecular Pathology and Metabolic Disease, Faculty of Pharmaceutical Sciences, Tokyo University of Science, 2641 Yamazaki, Noda-shi, Chiba 278-0022, Japan.
5) Molecular Profiling Research Center for Drug Discovery, AIST Tokyo Waterfront Bio-IT Research Building 2-4-7 Aomi ,Koto-ku, Tokyo 135-0064, Japan
6) Division of Supportive Care Research, National Cancer Center Research Institute, 5-1-1 Tsukiji, Chuo-ku, Tokyo 104-0045, Japan

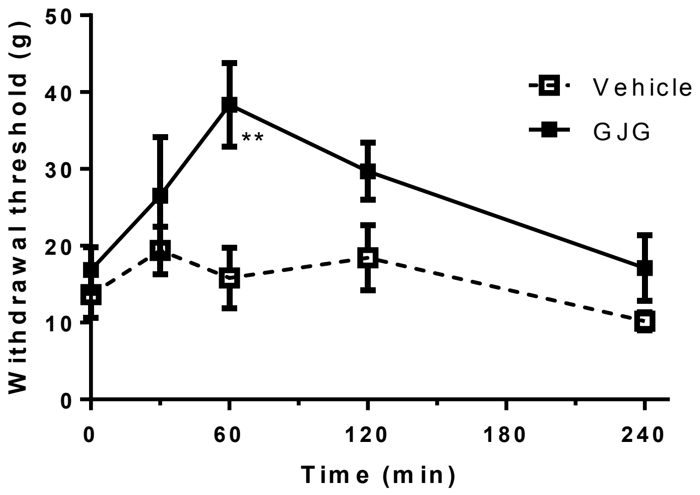

Supplemental Figure S1. Amelioration of mechanical allodynia by GJG in a rat CCI-neuropathic pain model. Fourteen days after CCI in the left hind paw, 2 g/kg GJG or its vehicle were orally administered and the pressure threshold of paw withdrawal was measured at each time-point. GJG significantly increased the withdrawal threshold 60 min after administration. Data shown represent mean +/- SEM (n = 4 in vehicle, n = 5 in GJG). ** $P < 0.01$; by two-way ANOVA with Bonferroni's correction.

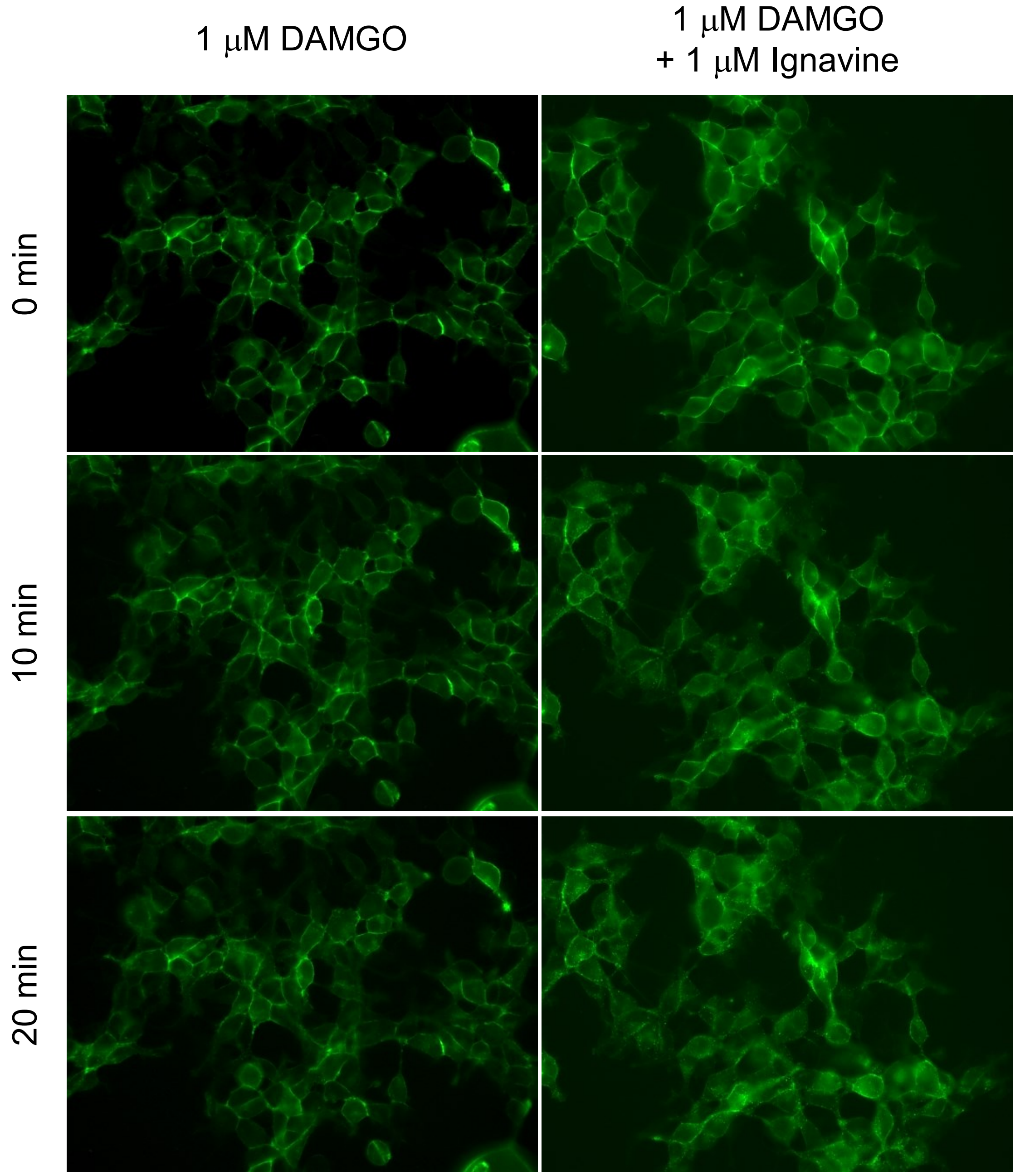

Supplemental Figure S2. All field views in receptor internalization assay. 1 μM DAMGO induced receptor internalization 20 min after application. Receptor internalization was observed 10 min after application of 1 μM DAMGO with 1 μM ignavine.

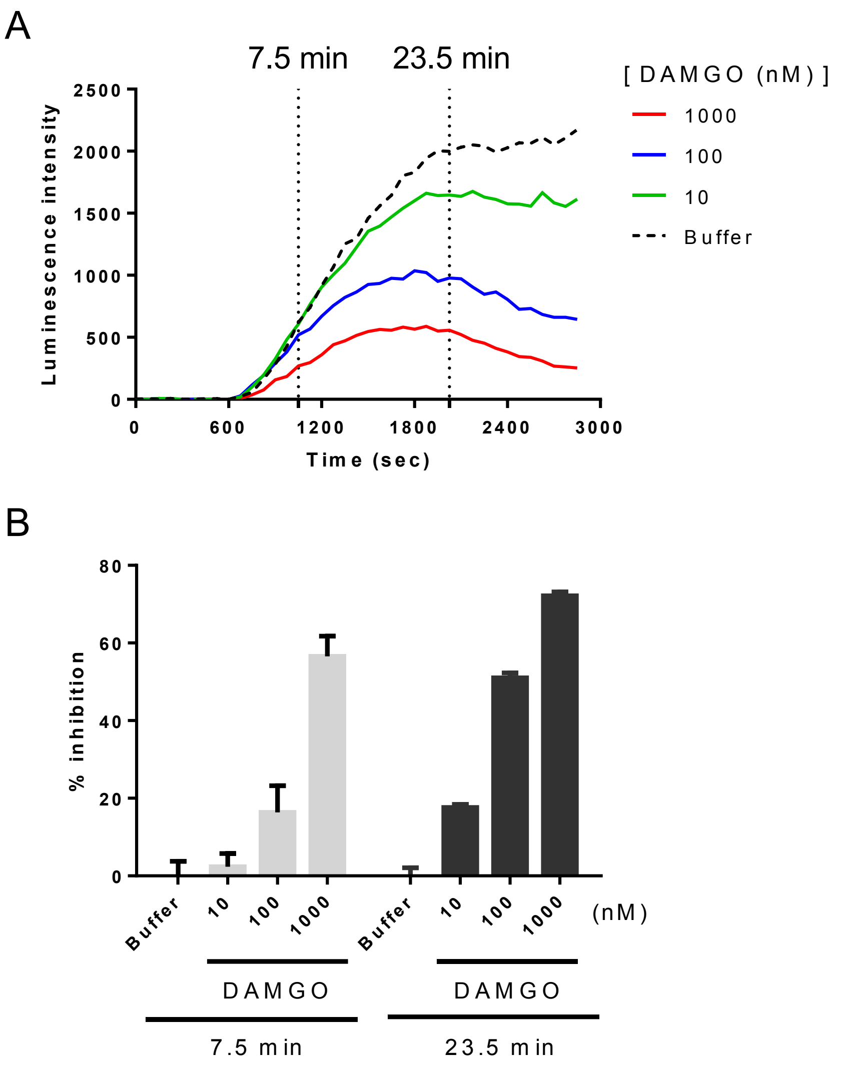

Supplementary Figure S3. Intracellular cAMP assay using recombinant human MOR and a GloSensor™ protein-expressing cell line. The cells were treated with DAMGO at 0 sec. Then, forskolin was added at 10 min (600 sec). **A** The amount of intracellular cAMP was traced by luminescence intensity. DAMGO inhibited the increase in intracellular cAMP in a dose-dependent manner. **B** % inhibition was calculated at 7.5 and 23.5 min after forskolin stimulation. Data shown represent mean (n = 3).

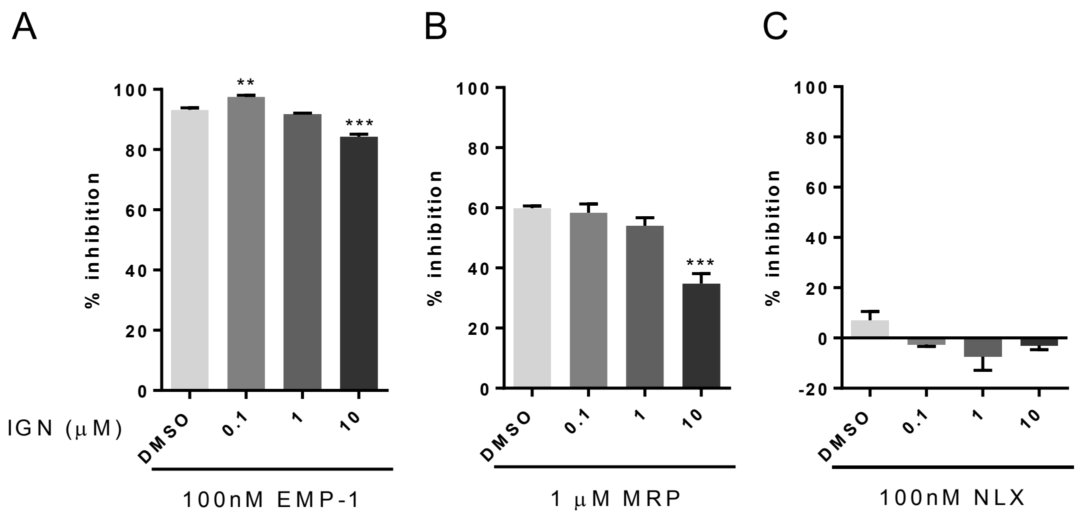

Supplementary Figure S4. Intracellular cAMP assay. The effect of ignavine on MOR expressing cell line in the presence of 100 nM endomorphin-1 (EMP-1, **A**), 1 uM morphine (MRP, **B**) and 100 nM naloxone (NLX, **C**) was evaluated. % inhibition was calculated from the data obtained at 23.5 min after forskolin application. Data represent the mean +/- SEM (n = 3). ** $P < 0.01$; *** $P < 0.001$ by Dunnett's test vs. vehicle.

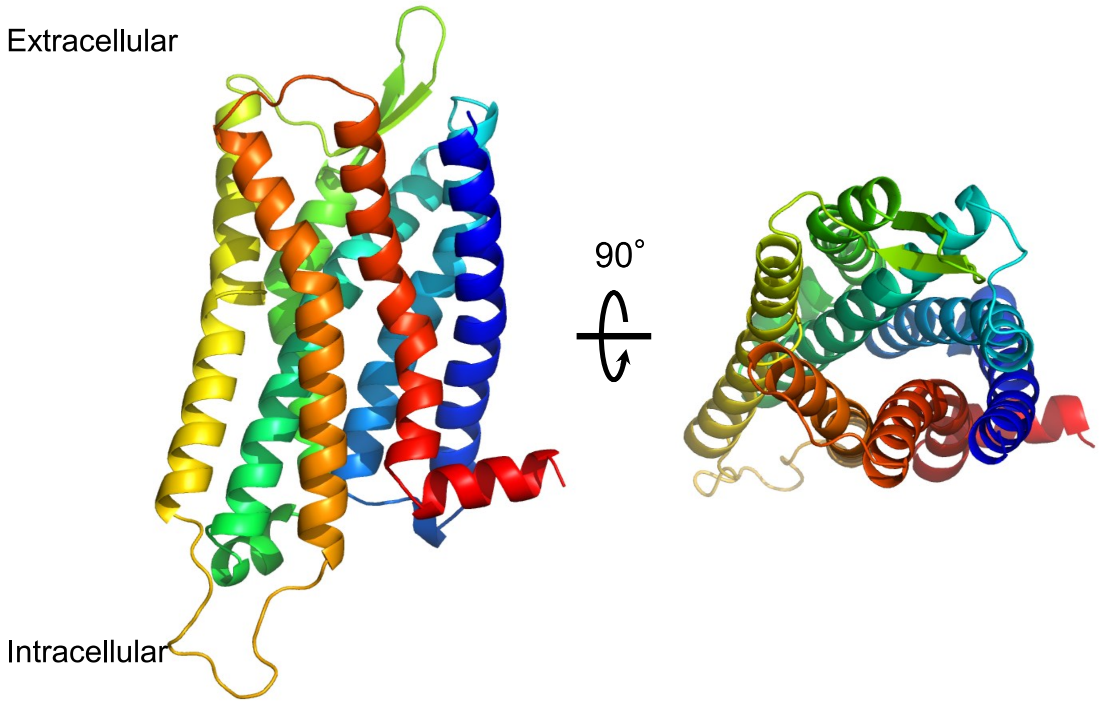

Supplementary Figure S5. The 3D structure of the complete human MOR homology model. The structure of the complete human MOR viewed from a position parallel to the membrane (left) and from the extracellular side (right). Each of the transmembrane helices are represented in different colors.
